# Performance of high resolution (400 m) PM_2.5_ forecast over Delhi

**DOI:** 10.1038/s41598-021-83467-8

**Published:** 2021-02-18

**Authors:** Chinmay Jena, Sachin D. Ghude, Rajesh Kumar, Sreyashi Debnath, Gaurav Govardhan, Vijay K. Soni, Santosh H. Kulkarni, G. Beig, Ravi S. Nanjundiah, M. Rajeevan

**Affiliations:** 1 Indian Institute of Tropical Meteorology, Ministry of Earth Sciences, Pune, India; 2 National Center for Atmospheric Research, Boulder, CO 80301 USA; 3 Department of Atmospheric and Space Sciences, Savitribai Phule Pune University, Pune, India; 4 India Meteorological Department, Ministry of Earth Sciences, New Delhi, India; 5 Centre for Development of Advanced Computing, Pune, 411 008 India; 6 Centre for Atmospheric and Oceanic Sciences, Indian Institute of Science, Bengaluru, 560012 India; 7 National Center for Medium Range Weather Forecasting, Ministry of Earth Sciences, Noida, UP India; 8 Ministry of Earth Sciences, Prithvi Bhavan, Lodhi Road, New Delhi, 110003 India

**Keywords:** Atmospheric science, Environmental sciences

## Abstract

This study reports a very high-resolution (400 m grid-spacing) operational air quality forecasting system developed to alert residents of Delhi and the National Capital Region (NCR) about forthcoming acute air pollution episodes. Such a high-resolution system has been developed for the first time and is evaluated during October 2019-February 2020. The system assimilates near real-time aerosol observations from in situ and space-borne platform in the Weather Research and Forecasting model coupled with Chemistry (WRF-Chem) to produce a 72-h forecast daily in a dynamical downscaling framework. The assimilation of aerosol optical depth and surface PM_2.5_ observations improves the initial condition for surface PM_2.5_ by about 45 µg/m^3^ (about 50%).The accuracy of the forecast degrades slightly with lead time as mean bias increase from + 2.5 µg/m^3^ on the first day to − 17 µg/m^3^ on the third day of forecast. Our forecast is found to be very skillful both for PM_2.5_ concentration and unhealthy/ very unhealthy air quality index categories, and has been helping the decision-makers in Delhi make informed decisions.

## Introduction

Delhi, being the second most populated megacity in the world, faces a range of environmental challenges including adverse air pollution episodes particularly during the winter season^1–5^. Exposure of large fraction of the population to poor air quality, pose a higher health risk^6–10^. In recent years, particulate matter of aerodynamic diameter smaller than 2.5 µm (PM_2.5_) has dominated severe air pollution episodes, and been severely affecting daily life in Delhi^11,12^. Thus, managing air quality with practical mitigation options has emerged as one of the top priorities of the Government of India (GOI) without compromising the current and projected growth in the overall economy, infrastructure development, industries, and service sectors.

The GOI is committed to enforce policy-driven measures to reduce the pollutant emissions. The National Clean Air Program (NCAP) initiated by the GOI targets significant reduction of surface PM_2.5_ concentrations by the year 2024. A Graded Response Action Plan (GRAP) has been designed for the National Capital Region (NCR) that allows pollution control authorities to reduce the magnitude of predicted air pollution for different air quality index (AQI) categories by imposing temporary control measures. Activation of different GRAP measures requires information of forthcoming extreme air pollution episodes so that effective temporary control measures can be identified early and implemented in advance. Therefore, GOI mandate required the Ministry of Earth Science (MoES) to develop an operational high-resolution air quality forecasting system for the NCR. In response to this mandate, MoES institutions namely the, Indian Institute of Tropical Meteorology (IITM) and India Meteorology Department (IMD), has developed a first very high-resolution (400 m) chemical weather forecasting capability in mutual collaboration with U.S. National Centre for Atmospheric Research (NCAR). The initial capability was developed for Delhi during 2018 based on WRF-Chem model at horizontal grid-spacing of 10 km and 2 km^13^. The first version of the forecasting system assimilated the Moderate Resolution Imaging Spectro-radiometer (MODIS) aerosol optical depth (AOD) retrievals and significantly improved air quality decision-making activity by reducing biases in 72-h PM_2.5_ forecasts to a greater extent^14^. However, that system struggled to predict the absolute PM_2.5_ levels during very acute air pollution episodes characterized by surface PM_2.5_ mass concentrations greater than 350 µg/m^3^^13,14^.

To further enhance the air quality decision-making activity by accurately predicting the PM_2.5_ concentrations during acute air pollution episodes, the modelling framework was further extended to produce PM_2.5_ forecast at much higher grid-spacing of 400 m. This new framework is envisaged to make the neighborhood scale air-quality management initiatives (e.g. GRAP) more effective. The finer grid-spacing would also represent the emission-sources in a much robust and realistic manner. To the best of our knowledge, none of the operational centres are currently providing short-term operational air quality forecasts at a spatial scale of 400 × 400 m^2^ covering approximately 50 km^2^ areas. This is the first attempt to develop and evaluate the performance of PM_2.5_ forecasts in a highly polluted environment using integration of dynamical models with chemical data assimilation. This high-resolution forecasting system consists of a newly developed high-resolution (400 m grid-spacing) emission inventory for Delhi, assimilation of MODIS AOD and in-situ observations of surface PM_2.5_ concentrations from a dense air quality monitoring network in Delhi to improve regional and local initialization of aerosols, ingests near real-time fire emissions, and applies a high-resolution dynamical downscaling into the WRF-Chem model. The PM_2.5_ forecasts at 400 m grid-spacing were made operational in October 2019.

Here, we provide a brief description of this newly developed operational high-resolution forecasting system, highlight the impact of data assimilation, and evaluate the quality of the PM_2.5_ operational forecast for the latest winter season in Delhi. We show that the forecast falls within the expected uncertainties and, is therefore suitable for issuing timely warnings to the residents of Delhi and NCR.

## Data and methods

The architecture of the air quality early warning system is shown in Fig. S1. The major components of the system are (a) numerical air quality predication model WRF-Chem, (b) Gridpoint Statistical Interpolation (GSI) based three-dimensional variational (3D-var) data assimilation system, (c) observational data and emission pre-processers, (d) WRF-Chem output post-processor, and (e) a public dissemination system (https://ews.tropmet.res.in/). The system starts every day at 5:30 pm Indian Standard Time (IST, which is 5:30 h ahead of the UTC), finishes the forecasts and post-processing overnight and disseminates the air quality forecast products in the morning for the next 72 h.

The core of the forecasting system consists of the regional WRF-Chem Version 3.9.1 configured in a three domain set-up with the outer domain covering northern part of the Indian subcontinent at a horizontal grid-spacing of 10 km × 10 km, the second domain covering the NCR and neighboring states at 2 km × 2 km grid-spacing, and the innermost domain covering Delhi at 400 m × 400 m grid-spacing (Fig. S2). The meteorological initial and boundary conditions are based on the analysis and forecast product (Ensemble-Kalman filtering) produced by the IITM-Global Forecasting System (IITM-GFS, T1534) spectral model at 12.5 km grid spacing available every three hours. The outermost domain (D1) is provided with the six-hourly chemical boundary conditions from the Model for Ozone and related Chemical Tracers version 4 (MOZART-4) 10-year climatology. However, the chemistry output from the outer domain (D1) is dynamically used to provide chemical boundary conditions for the first inner domain (D2) every three-hour. Similarly, the output from the domain D2 is dynamically used to provide boundary conditions for the final 400 m domain (D3). The physical and chemical parameterizations used in the model are listed in Supplementary Table ST1. We use MOZART-4 gas-phase chemistry linked to Goddard Chemistry Aerosol Radiation and Transport (GOCART) aerosol scheme (MOZCART) to represent gas-phase chemistry and aerosol processes in our system. In the GOCART scheme, aerosol species namely nitrates and secondary organic aerosols are missing and are a source of uncertainty in the estimated PM_2.5_. Both MOSAIC (Model for Simulating Aerosol Interactions and Chemistry) and GOCART have been found to underestimate the observed wintertime PM_2.5_ mass concentrations in Delhi with MOSAIC showing superior performance^15^. However, the computationally efficiency of GOCART compared to MOSAIC combined with the positive impact of chemical data assimilation motivated us to use GOCART in operations.

We used three-dimensional variational (3D-VAR) method of the community Grid-point Statistical Interpolation (GSI) system Version 3.5. The 3D-VAR scheme blends the information from the satellite AOD and surface PM_2.5_ observations, and iteratively minimizes a cost function $\mathbf{J}$ that depends on observation and background error covariance matrices as defined in Eq. (1).

1 $${\text{J}}({\mathbf{x}}) = \frac{1}{2}({\mathbf{x}} - {\mathbf{x}}_{{\mathbf{b}}} )^{{\text{T}}} {\mathbf{B}}^{{ - 1}} \left( {{\mathbf{x}} - {\mathbf{x}}_{{\mathbf{b}}} } \right) + \frac{1}{2}({\text{H}}({\mathbf{x}}) - {\mathbf{y}})^{{\text{T}}} {\mathbf{R}}^{{ - 1}} \left( {{\text{H}}({\mathbf{x}}) - {\mathbf{y}}} \right)$$

where $\mathbf{x}$ represents the state vector that contains aerosol chemical composition and other meteorological variables required for computing AOD, ${\mathbf{x}}_{\mathbf{b}}$ represents the “a priori” information about **x** and is referred to as background state, **B** is the background error covariance (BEC) matrix, H is the forward operator that transforms WRF-Chem aerosol chemical composition to AOD following Liu et al.^16^, $\mathbf{y}$ represents the MODIS AOD retrievals, and ***R*** is the observation error covariance matrix. BEC statistical parameters are calculated using two 24-h WRF-Chem forecast initialized at 00 z with different meteorological conditions, anthropogenic emissions, and biomass burning emissions to account for the uncertainties in meteorology, anthropogenic, and biomass burning emissions^17^. Observations from MODIS overpasses at 10:30 and 1:30 Local Time (L.T.) at 10 km grid spacing and hourly mean surface PM_2.5_ observations from the 37 monitoring stations (Fig. S3) across Delhi are collected at the initial time (0900 UTC) of each forecast cycle. GOCART has sixteen aerosol species and all of them are adjusted in response to AOD assimilation whereas only the species contributing to PM_2.5_ are adjusted in response to surface PM_2.5_ assimilation.

Parameters for modeling the background error covariance (BEC) matrix are estimated using the National Meteorological Center (NMC)^18^ method of a community Generalized Background Error (GEN_BE). The NMC method uses the difference between two forecasts valid at the same time to model the BEC matrix. Here, two 24-h WRF-Chem forecasts initialized at 00 Z with different meteorological, anthropogenic emissions, and biomass burning emission inputs are generated every day from January to December, 2018. Different anthropogenic and biomass burning emissions are used to incorporate uncertainties in anthropogenic and biomass burning emissions in the BEC. Meteorological uncertainties are represented by driving the WRF-Chem forecasts with the Global Forecast System (GFS) and Era-Interim reanalysis datasets. We assume 100% uncertainties in both the anthropogenic and biomass burning emissions following inter-comparison of different anthropogenic^19^ and biomass burning^20^ emission inventories. A total of 30/31(28 in February) pairs of forecasts are generated at 09 UTC (corresponding to MODIS Aqua overpass time) every month and supplied to the GEN_BE for calculation of the BEC statistical parameters in three stages. The first stage reads these paired WRF-Chem forecasts and stores the difference between the two forecasts in a binary file per day in every month. The second stage removes the temporal mean from the differences generated in stage 1 and stores the perturbations around the mean in another set of binary files for each day. The third stage uses the perturbation files from the second stage to calculate the statistical parameters, i.e., variance, horizontal length scale, and vertical length scales which are then used to model the background error in GSI. The variance determines how much of the innovation (difference between the model and observations) becomes the analysis increment whereas horizontal and vertical length scales determine how the analysis increment influences the neighboring grids horizontally and vertically. Background error variances for all the species are the highest in the boundary layer, i.e., below 3 km which reflects larger uncertainties in aerosols near the surface except for sea-salt for which standard deviation values similar to boundary layer are also seen in the free troposphere. The variance is the highest for OC_2_. The horizontal and vertical length scales are within 1–3 grid points.

Since GOCART is a bulk aerosol model and does not simulate size distribution of aerosols, the forward operator H, i.e., Community Radiative Transfer Model (CRTM) assumes that each aerosol chemical component follows a log-normal size distribution. The effective radii are assumed to be 0.242 µm for sulfate, 0.087 µm for organic carbon, and 0.036 µm for black carbon in dry air with the standard deviation of 2.03 µm, 2.2 µm, and 2.0 µm, respectively. The CRTM uses the same effective radii for dust and sea-salt aerosols that are used in GOCART, i.e., 0.3 µm, 1.0 µm, 3.25 µm, and 7.5 µm for the four bins of sea-salt aerosols and 0.73 µm, 1.4 µm, 2.4 µm, 4.5 µm, and 8.0 µm for five bins of the dust aerosols. The standard deviation of sea-salt and dust aerosols are assumed to be 2.03 µm and 2.0 µm, respectively. The hygroscopic growth of hydrophilic aerosols (i.e., sulfate, sea-salt, hydrophilic components of organic carbon and black carbon) is also calculated as a function of relative humidity from the pre-computed look-up tables. Refractive indices for different dry aerosol components are based on the Optical Properties of Aerosols and Clouds (OPAC) database^21^ and that of water is based on Hale & Querry et al.^22^. After calculating size distribution and refractive indices, Mie theory is employed to calculate mass extinction coefficient which is then multiplied with aerosol columnar mass concentrations to obtain AOD. The model AOD is compared with MODIS AOD at the satellite retrieval location and the difference between model and MODIS AOD is minimized using a preconditioned conjugate gradient method. The changes in AOD due to assimilation are translated back to the aerosol chemical composition using the adjoint of the forward operator which is described along with mass extinction coefficients at 550 nm, and density of different aerosol types in Sect. 3.4 of Liu et al.^16^.

We assimilate the near-real time MODIS AOD retrievals in WRF-Chem that are available 3 h after the satellite overpass. The observation errors are specified as (0.03 + 0.05*AOD) and (0.05 + 0.15*AOD) over ocean and land, respectively. The error in surface PM_2.5_ observation is specified as 1.5 µg/m^3^. MODIS AOD from both Terra and Aqua satellites as well as surface PM_2.5_ observation is assumed to be available for assimilation at 09 UTC. MODIS near real-time retrievals are available with a latency of 3 h, which means that they become available at about 16:30 IST. Downloading and processing of the near-real time MODIS AOD retrieval takes about 15 min in the operational forecasting set-up. MODIS AOD retrievals are not available during cloudy conditions and our system relies on the in situ PM_2.5_ observations for improving the initial conditions during those situations. However, this problem is more prevalent during the monsoon season but not as much during winter which is the focus here.

Every day, the chemical fields are initialized from the previous day’s WRF-Chem forecast, aerosol fields are updated through assimilation, and meteorology is refreshed using the IITM-GFS forecast. The first version of our forecasting system used the EDGAR-HTAP emission inventory to represent anthropogenic emissions. We have developed two more anthropogenic emissions to test the sensitivity of our forecasting setup to input anthropogenic emissions. First, we scaled 2010 EDGAR-HTAP emissions for the outer (D1) and the first inner (D2) domains to 2019 using scaling factors given in Venkataraman et al.^23^. For Delhi itself, i.e., the innermost domain (D3), we used 400 m High-resolution Delhi Emission Inventory (HrDEI) for the year 2018 developed under the MoES (Fig. S4) System of Air Quality and Weather Forecasting and Research (SAFAR) project (http://safar.tropmet.res.in/source.pdf). For D1 and D2, the original 400 m emissions were processed using a mass-conserving approach and mapped to match the 10 km and 2 km grid spacing so that the total mass emitted is same before and after re-grinding. We adopted diurnal variation in emissions from a recent study by Govardhan et al.^24^. The Model of Emissions of Gases and Aerosols from Nature (MEGAN)^25^ is used to calculate biogenic emissions online within the model. Dust emissions are based on the online Atmospheric and Environmental Research/Air Force Weather Agency (AER/AFWA) scheme^26^. Our recent study shows that post-monsoon biomass burning emission significantly affects the air quality in Delhi^26^. Therefore, an accurate representation of fire emissions is essential for the accuracy of the forecast. Most of the near real-time biomass burning emission estimates are available with a time lag of one day and thus operational air quality forecasts are forced to assume persistent fire emissions over the forecast cycle. Here, we have developed a pre-processor based on the high-resolution Fire INventory from NCAR (FINN)^20^ to derive fire emissions for the forecast day instead of using the estimates from a previous day. First, we developed a historical daily gridded (at the model grid spacing) data set of fire emissions from 2002 to 2018. Second, daily fire location information is obtained from the near real-time MODIS-C6 active fire data from FIRMS (https://firms.modaps.eosdis.nasa.gov/). Finally, fire activity obtained in the second step is compared with the fire activity in the historical dataset and fire emissions corresponding to the best match between present-day and past fire activity are used in each grid cell.To include the fire emissions for the next two days, we calculated the historical fire frequency for each day and for each model grid based on a 10-year gridded data set. We include the fire emissions only in those grid cells where confidence level of fire count is more than 50%.

Quality controlled PM_2.5_ observations are obtained in near real-time from the 37 air quality monitoring stations operated by the Central Pollution Control Board (CPCB), Delhi Pollution Control Committee (DPCC), and the IITM. Details about the calibration of the instruments and quality controlled procedure can be seen here https://cpcb.nic.in/quality-assurance-quality-control/. In addition to the automated CPCB quality control, we apply additional filters to remove spurious observations by rejecting, PM_2.5_ measurements above 1500 µg/m^3^, and those corresponding to instrument malfunction^14^. The details of these monitoring locations are given in the supplementary material (Table ST3), and the geographical locations are shown in Fig. S3. A variety of widely used statistical evaluation metrics such as mean bias (MB), Pearson’s correlation coefficient (r), normalized mean bias (NMB), normalized mean fractional error (NMFE)^27,28^ are used to evaluate the performance of PM_2.5_ forecasts.

## Results

### Influence of assimilation and high-resolution emissions on PM_2.5_ forecast

**Figure 1:**
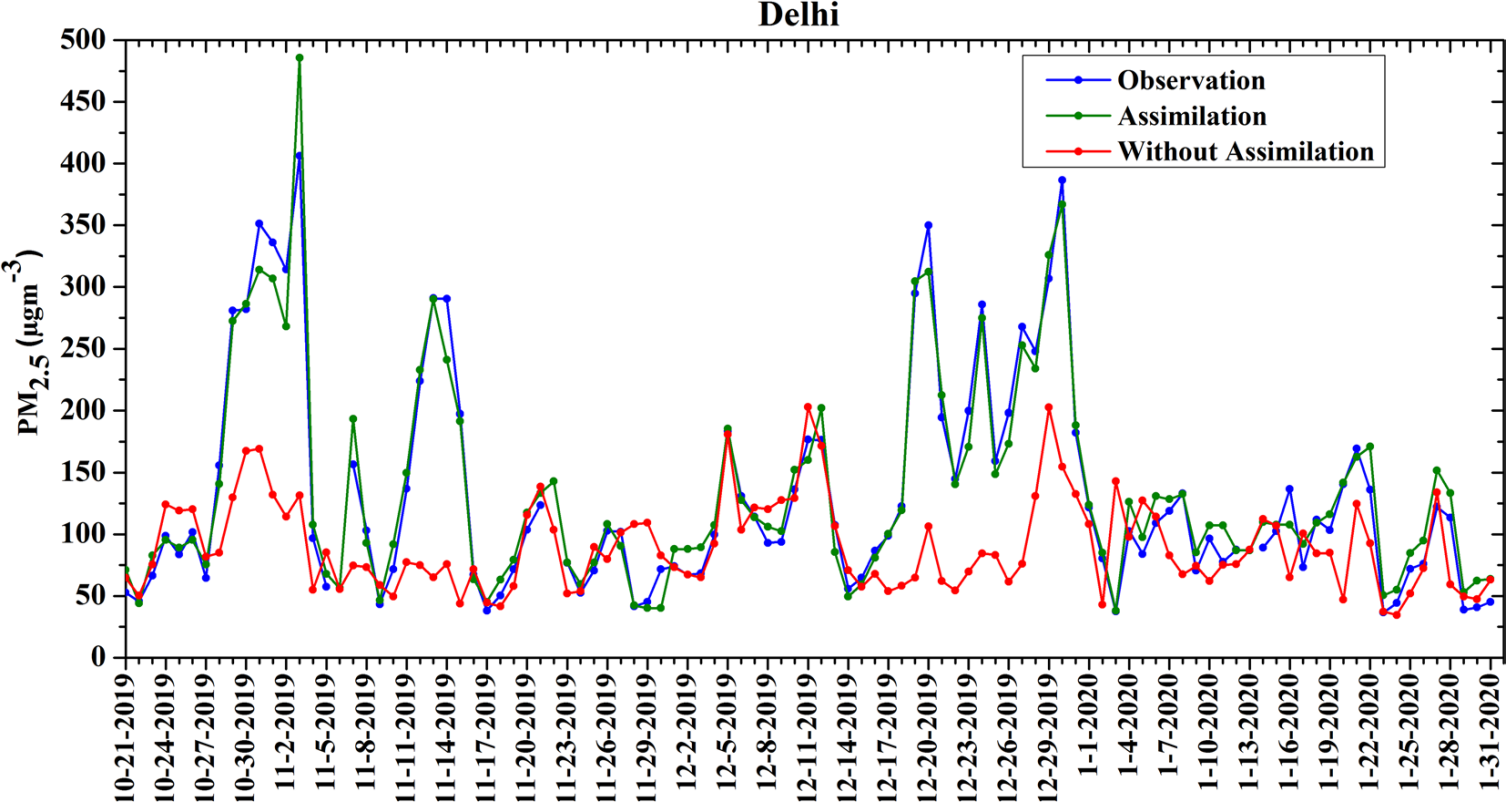
Averaged surface PM_2.5_ simulated by the model before (red) and after (green) assimilation at 0900 UTC assimilation cycle and its comparison with observed mean PM_2.5_ over Delhi during 21 October 2019 to 01 February 2020. The model output is averaged over the observational locations across Delhi.

To understand how much the assimilation of surface PM_2.5_ and satellite AOD observations changes the WRF-Chem PM_2.5_ at the initialization time every day, modeled surface PM_2.5_ concentrations averaged at all the stations before and after assimilation are compared with the observations at 09:00 UTC (Fig. 1). Before assimilation, the model significantly underestimates the observed PM_2.5_ mass concentrations and assimilation pushes the initial PM_2.5_ concentrations in the model very close to the observations. The average observed PM_2.5_ is about 133 ± 88 µg/m^3^, whereas simulated PM_2.5_ concentrations before and after assimilation are estimated as 90 ± 36 µg/m^3^ (mean bias = − 32%) and 135 ± 83 µg/m^3^ (mean bias =  + 1%), respectively. This indicates that assimilation improves the initial condition for PM_2.5_ by ~ 45 µg/m^3^ (about 50%).

We performed emission sensitivity simulations by keeping EDGAR emissions for the entire model domain and then replacing the Delhi region with high-resolution Delhi emission inventory (HrDEI). We find that PM_2.5_ mass concentration simulated by the EDGAR-HTAP only emissions largely under-predicted (bias = − 52%) the observed PM_2.5_ mass concentration in Delhi, whereas the original HrDEI 400 m resolution inventory over-predicted the observations by 36% during the winter season (Fig. S5). An additional simulation in which HrDEI emissions are reduced by 40% showed better agreement with observations (bias = 3%). Therefore, in the forecasting setup, we choose to reduce the HrDEI emissions by 40% over the Delhi region.

We analyze the effects of inclusion of such high-resolution emissions on the simulated PM_2.5_ concentrations by comparing the simulated PM_2.5_ concentrations with the observations from three sensitivity simulations (Fig. S6). These sensitivity simulations are designed to delineate the relative benefits of finer grid-spacing and the improved emission inventory in improving the PM_2.5_ forecasts. The first simulation uses EDGAR anthropogenic emissions in D1 (green line, Fig. S6); the second simulation uses HrDEI emissions mapped in D1 (blue line, Fig. S6); and the third simulation is performed with the new model set-up with HrDEI emissions at 400 m grid-spacing in D3 (red line, Fig. S6). All the three simulations capture the day-to-day variability in the observed PM_2.5_ mass concentrations very well but the first simulations with original EDGAR emissions fail to capture the observed peaks in PM_2.5_ concentrations. Upon just switching from the EDGAR emissions to the HrDEI emissions upscaled from 400 m to 10 km grid spacing, the model captured all the observed peaks in PM_2.5_ concentrations particularly after 17 Nov. Switching the grid-spacing from 10 km to 400 m, i.e., the third simulation does not improve the model performance further. The performance statistics for these three sensitivity simulations for hourly and daily mean of the simulated PM_2.5_ mass concentration for the first day is evaluated by examining the mean bias (MB), Pearson’s correlation coefficient (r), normalized mean fractional bias (NMFB) and normalized mean fractional error (NMFE) (Table ST2). We find that the PM_2.5_ mass concentration simulated by the model employing EDGAR emissions largely under-predicts the observed PM_2.5_ mass concentration in Delhi as indicated by higher values of MB, NMFB, and NMFE. On the other hand, the corresponding values for the model simulations involving HrDEI emissions are substantially better. These new simulations highlight that development of a 400 m resolution emission inventory is the primary driver of the improvement in air quality forecast rather than the model resolution during wintertime in Delhi. This suggests that 10 km grid spacing is resolving the processes controlling PM_2.5_ during this period in Delhi but similar analysis must be performed for other seasons and multiple years to determine if air quality forecasts at 10 km grid spacing driven by anthropogenic emissions upscaled from a 400 m emission inventory are of the same value as we found for this season.

### Performance of the PM_2.5_ and Air Quality Index (AQI) forecast

**Figure 2:**
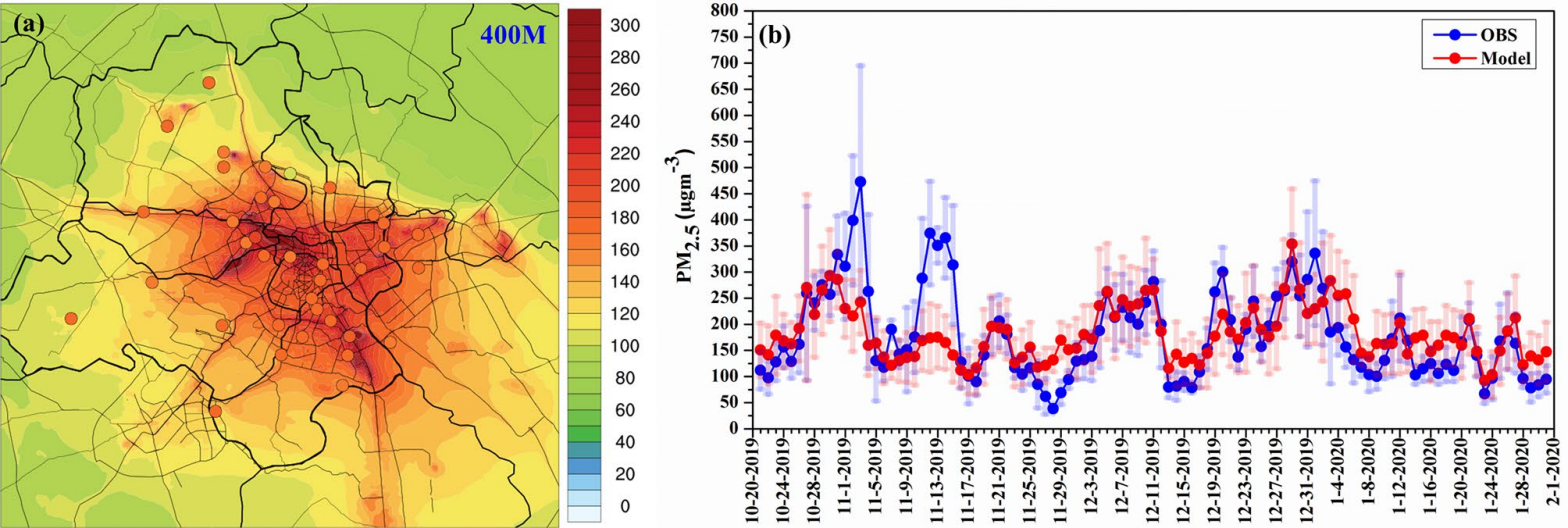
**(a)** Spatial distribution of averaged PM_2.5_ concentrations at 400 m horizontal grid-spacing (from day 1 forecast) overlaid with mean PM_2.5_ observed at different monitoring stations across Delhi during 21 October 2019 to 01 February 2020, **(b)** Comparisons between daily mean PM_2.5_ forecast (red) and daily mean PM_2.5_ observations (blue) over Delhi during 21 October 2019 to 01 February 2020 (vertical bar shows the standard deviation).

The air quality forecast verification period (21 October 2019 to 02 February 2020) selected here was dominated by the large-scale open biomass burning in October and first half of November followed by wintertime stable meteorological conditions which are conducive for build-up of PM_2.5_ pollution in Delhi. An example of the spatial distribution of average PM_2.5_ concentration from day 1 forecast is shown in Fig. 2a. As expected, D3 resolves emission sources in Delhi much better than the 10 km and 2 km domains (Fig. S7). At finer (400 m) grid-spacing, PM_2.5_ hotspots associated with the industrial, dense residential, major traffic junctions, and high-density vehicular traffic roads can be clearly distinguished (Fig. 2a). Daily air quality forecasts (Fig. 2b) reproduce the daily variation of mean observations quite well in the NCR. However, model performance at individual monitoring stations (supplementary Table ST3) shows that the NMB varies from − 46% to 85% among the 37 stations located across the NCR. Out of 37 stations, 24 stations (65% of stations) show NMB within ± 30%, and 5 stations (13% of stations) show NMB exceeding 50%.

**Figure 3:**
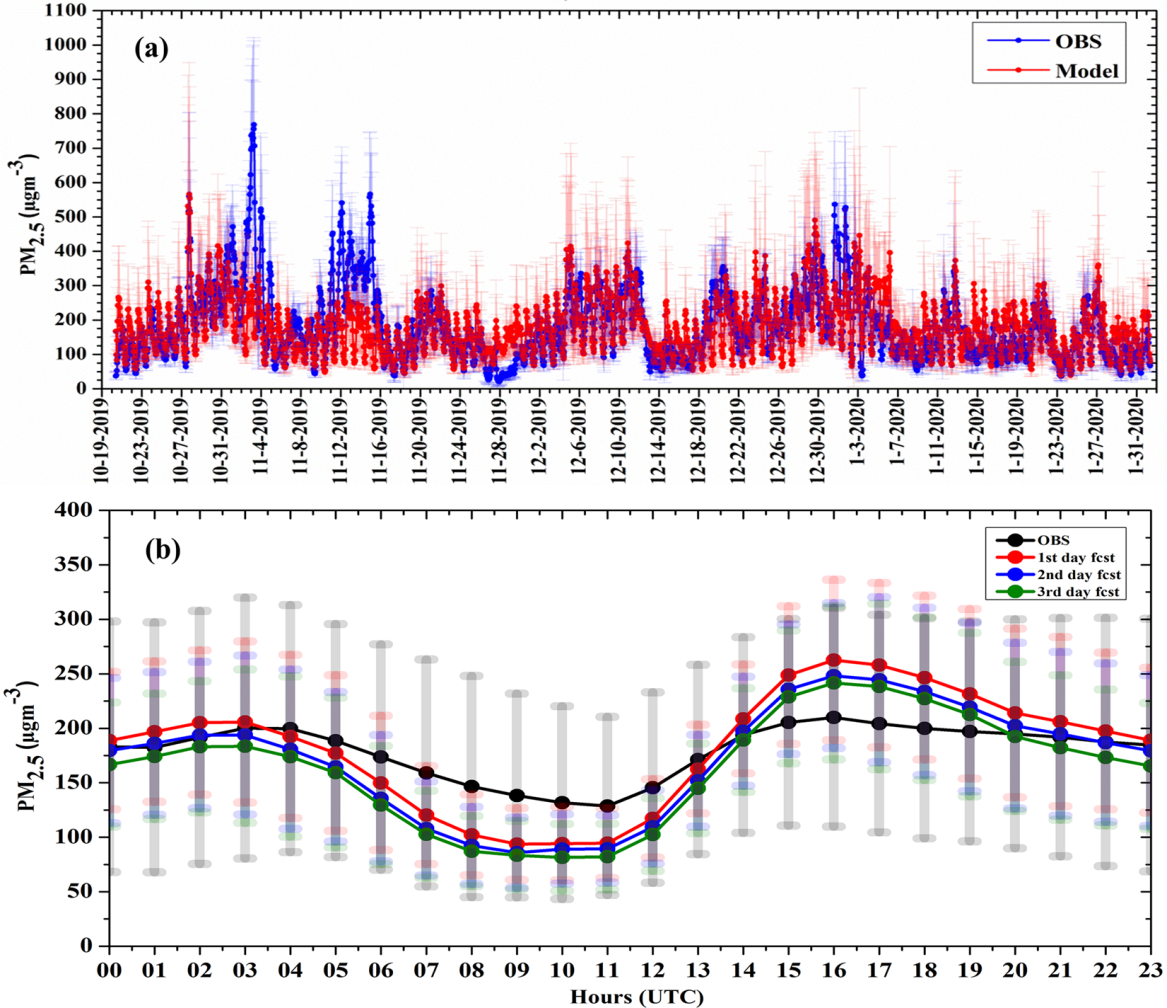
(**a**) Comparisons between hourly mean PM_2.5_ forecast (red) and hourly mean PM_2.5_ observations (blue) on day one forecast at 400 m horizontal grid spacing over Delhi during 21 October 2019 to 01 February 2020 (vertical bar shows the standard deviation) and (**b**) diurnal plots of hourly mean PM_2.5_ observations and hourly mean PM_2.5_ forecast on day one (red), day two (blue) and day three (green).

To assess the ability of the model in capturing NCR scale variability in PM_2.5_ concentrations, we compared the hourly time series of observed and modeled PM_2.5_ averaged at all the observation sites. The comparison based on the first day of forecast from D3 is depicted in Fig. 3a. The forecast captures the temporal variability in PM_2.5_ observation quite well on most of the days (Fig. 3a). On two occasions, 2–4 November and 12–16 November, the model failed to capture extremely high PM_2.5_ values observed in the NCR. However, very-poor AQI category pollution events observed on 20–22 November, and very-poor to severe AQI category events observed on 5–12 December, 19–22 December, 19–21 December, 29 December 2019–5 January 2020 were captured very well by our forecasting system. The sudden drop in PM_2.5_ levels (e.g., on 14 December, 7 January, and 24 January, etc.) followed by the very-poor to severe AQI category events were also captured very well by the model. The temporal variation in observed PM_2.5_ over Delhi are driven mainly by the frequent large scale open biomass burning^29^ and wintertime synoptic-scale meteorological condition in combination with the large anthropogenic emissions in Delhi itself. The ability of the model to capture this variability indicates that the forecast system has excellent skills in issuing PM_2.5_ forecasts associated with urban pollution and regional-scale events. The seasonal-mean diurnal variations of the observed and modeled PM_2.5_ mass concentration are compared in Fig. 3b. The model captures the bi-modal behavior of the observed PM_2.5_ mass concentration arising mainly from the interactions between increased emissions during rush-hours of the day and the atmospheric boundary-layer processes^30^. Even on day 3 of the forecast (green line, Fig. 3b), the model captures hourly variations in PM_2.5_ concentrations which highlights the usefulness of our air-quality forecast on the 72-h horizon frame.

The performance statistics for hourly mean PM_2.5_ forecast for the first, second and third day is evaluated by examining MB, RMSE, r, NMFB, and NMFE (Table 1). Following Morris et al.^31^, we have adopted three levels of performance criteria for fractional bias and error to evaluate the forecast performance (Supplementary Table ST4). The MB statistics shows that the model slightly overestimates the observed PM_2.5_ concentrations by 2.5 µg/m^3^, but changes behavior to underestimation by about − 9 µg/m^3^and − 17 µg/m^3^on the second and third day of the forecast, respectively. Table 1 reveals that PM_2.5_ forecast performed close to excellent criteria on day 1 as the NMFB and NMFE are within 1.3% and 36.3%, respectively. The model performance is good on day 2 and day 3 with fairly low NMFB (< ± 10%), and NMFE is within ± 40%. Performance statistics on daily mean PM_2.5_ time series show excellent performance on all the three days of forecast since NMFB and NMFE were within ± 10% and 29%, respectively. The correlation coefficient (r) is around 0.5 for the hourly forecast and 0.6 for the daily mean forecast. It appears from Fig. S8 that there is a considerable scatter between observed and predicted PM_2.5_. The forecasts generally show a tendency to under predict the higher values and slightly over predict the lower values, consistent with the other studies^32,33^. Efforts are underway to investigate why this underestimation in higher values occurs and whether it is the result of errors in the meteorological, boundary condition, emission, or chemical factors.

**Table 1:** Performance statistics for mean PM_2.5_ forecast and skill score for different forecast AQI category over Delhi during 21 October 2019 to 01 February 2020.

Statistical performance					
State variables	Forecast day	400 meter			
		MB	NMFB (%)	NMFE (%)	r
PM_25__hourly	1st day	2.5	1.3	36.3	0.5
	2nd day	− 8.4	− 4.8	38.1	0.5
	3rd day	− 16.8	− 9.8	40.5	0.4
PM_25__daily	1st day	1.8	1.0	25.6	0.6
	2nd day	− 8.8	− 5.0	26.7	0.6
	3rd day	− 17.3	− 10.1	29.5	0.5
PM_25__AQI	1st day	21.7	6.5	16.5	0.7
	2nd day	10.4	3.1	16.5	0.6
	3rd day	0.3	0.1	17.8	0.5

Skill score for AQI					
AQI range	Forecast Day	FAR	POD	CSI	Accuracy
Unhealthy (poor, very-poor, severe)	1st day	0.11	1.00	0.88	0.88
	2nd day	0.09	0.99	0.90	0.90
	3rd day	0.09	0.98	0.88	0.88
Very unhealthy (very poor, severe)	1st day	0.28	0.98	0.70	0.72
	2nd day	0.25	0.94	0.71	0.75
Critical (severe)	3rd day	0.23	0.89	0.70	0.74
	1st day	0.35	0.34	0.29	0.82
	2nd day	0.15	0.35	0.33	0.85
	3rd day	0.25	0.21	0.19	0.82

The main objective of developing this high-resolution forecasting system is to provide timely air quality alerts to the residents of NCR, particularly for the Poor to Severe AQI category. As per the CPCB guidelines, AQI category is classified as poor for the AQI range 201–300, very-poor for the AQI range 301–400, and severe for the AQI range 401 and above. Therefore, it is essential to evaluate the applicability of the system to simulate the correct AQI values, as these are disseminated to the general public rather than the actual PM_2.5_ mass concentrations. Therefore, hourly AQI values for PM_2.5_ based on 24-h PM_2.5_ standard was calculated based on National Ambient Air Quality Standard (NAAQS), and break-point concentration suggested in the CPCB notification (see Supplementary Table ST5). Table 1 shows that the magnitude of MB for overall AQI values (0–500) was slightly higher (about 22 units) on the first day of forecast compared to MB observed on the second day (10 unit) and third day (< 1 unit). However, following the Morris et al.^31^ criteria, the AQI forecast on all three days showed “excellent” performance, since NMFB and NMFE are within ± 6% and 18%, respectively. The statistical performance (Supplementary Table ST6) indicates that the forecast over-predicts the poor air quality AQI category by about 22% on day one and by about 19% and 16% on days two and three, respectively. AQI forecast on all the three days in the poor AQI category performed good, since NMFB and NMFE are within ± 22% and 22%, respectively. For the very-poor AQI category, the AQI forecast performed excellent on all three days (NMFB < 5% and NMFE < 9%). In comparison, the forecasting system under-predicts the severe AQI category by about 14% on day one and by about 17% and 21% on days two and three, respectively. On the first day, the AQI forecast for the severe AQI category performed excellently (NMFB < 14% and NMFE < 17%), while it performed reasonably well on days two and three (NMFB < 21% and NMFE < 21%). Overall, the forecast falls within the expected uncertainties and, therefore, is suitable to help decision-makers make informed decisions.

### Skill score for categorical AQI forecast

To assess the skill of real-time forecast, i.e., whether the forecast will fall in unhealthy (AQI > 201), or very-unhealthy (AQI > 301) or critical category (AQI > 401), false alarm rate (FAR), probability of detection (POD) or hit rate, critical success index (CIS) and accuracy, are calculated according to Kang et al.^34^ and Eder et al.^33^. Supplementary Table ST7 describes the equations used to calculate the skill score of the categorical AQI forecast. Table 1 presents the skill score for the unhealthy, very-unhealthy, and critical categories of AQI for the winter season. The forecast accuracy for unhealthy category (i.e., forecast that correctly predicted the unhealthy or no-unhealthy AQI) is estimated to be > 88% on all the three days. The skill score for the POD and CSI is relatively promising with a value greater than ~ 0.9, which indicates that the model has good accuracy in predicting the unhealthy air quality conditions with respect to a total number of the observed air quality hours. Also note that FAR is quite low (~ 10%) for the unhealthy category, which indicates that the performance of the real-time high-resolution forecast was excellent for both unhealthy category and non-unhealthy category of air quality. For the very-unhealthy category, the POD score (> 0.9) is excellent, but CSI score is somewhat lower (~ 0.7), and the FAR score is a slightly higher (20–30%) compared to the unhealthy category on all three days of forecast. However, the skill scores overall indicate excellent performance for predicting air quality in the very-unhealthy category. On the other hand, the skill score shows moderate performance for the severe AQI category on all three days. Table 1 indicates that compared to the unhealthy and very-unhealthy category, the skill score for POD and CSI is low (< 0.35) on days one and two of forecast and further declines on day three. The accuracy of the forecast is about 80%, and the FAR score does not show a significant increase compared to the other two categories, which indicates the moderate skill of the forecasting system to predict the extremely high pollution events in NCR region. Some of the causes of unsuccessful prediction of accurate extremely high pollution events may include difficulties in simulating processes like boundary layer height, synoptic advection, and synoptic-scale conditions and choice of aerosol module parameterization. Our future studies will investigate the role of these processes in predicting the extreme PM_2.5_ pollution events in the NCR region.

## Conclusion

This study demonstrates the efficacy of a newly developed very high-resolution operational air quality forecasting system to issue timely warning to the residents of Delhi and NCR about forthcoming air pollution episodes. The system applies the WRF-Chem model in a dynamical downscaling framework and assimilates satellite AOD and surface PM_2.5_ observations for improving the initial conditions. Performance of the system is evaluated for both surface PM_2.5_ mass concentration and AQI categories to assist both local forecasters and air quality model developers. The assimilation of MODIS AOD and surface PM_2.5_ data, on an average, improves the initial condition for PM_2.5_ about by ~ 45 µg/m^3^ (~ 50%). Model evaluation shows that our forecasting system is capable of issuing reliable forecasts for Delhi during winter season both for PM_2.5_ concentration and AQI in particular for unhealthy and very-unhealthy AQI categories, within the expected uncertainties. On the other hand, verification statistics for the severe AQI category show moderate skill and require further improvement in the forecast. Although HrDEI emission inventory captures the spatial variability in emissions, it is desirable to have an accurate forecast at every individual station. The model showed moderate performance in capturing the accurate spatial distribution of PM_2.5_ across the NCR and requires further improvement. We recognize that the accuracy of the high-resolution emission inventory, choice of aerosol parameterization, chemical mechanism, and boundary layer parameterization will continue to be a challenge to forecast the PM_2.5_ at individual point monitoring locations in this region. Efforts are underway to explore the sensitivity of these parameters to the accuracy of location-specific PM_2.5_ forecast. For the first time, this system has also been used by the environmental pollution control authorities to make the decision on imposing/lifting the temporary restriction on construction activities and regulating the heavy vehicle inflow in Delhi region during the pollution/no-pollution events. This has significantly contributed in building-up the trust of the end-users and policy-makers for taking science-based well-informed decisions and actions for important public services in India.

## Supplementary Information

Supplementary Information.

## Data Availability

The 0.1° × 0.1° emission grid maps can be downloaded from the EDGAR website on https://edgar.jrc.ec.europa.eu/htap_v2/index.php?SECURE=_123 per year per sector. MODIS AOD retrievals used for assimilation can be downloaded from this site (https://earthdata.nasa.gov/). Observational data on PM_2.5_ measurements can be obtained from CPCB website on https://app.cpcbccr.com/ccr. The model data has been archived at Prithvi (IITM) super-computer and can be provided upon request to corresponding author.
