# Supplementary material for: Performance of high resolution (400 m) PM2.5 forecast over Delhi
Source: Sci Rep. 2021 Feb 18;11:4104. doi: 10.1038/s41598-021-83467-8 (PMC7892871; doi:10.1038/s41598-021-83467-8)
Supplement: Supplementary file 1 — Supplementary Information. [file 41598_2021_83467_MOESM1_ESM.docx]

**Performance of high resolution (400 m) PM_2.5_ forecast over Delhi**

Chinmay Jena^1*^, Sachin D. Ghude^1*^, Rajesh Kumar^2^, Sreyashi Debnath^1,3^, Gaurav Govardhan^1,7^ , Vijay K. Soni^4^, Santosh H. Kulkarni^5^, G. Beig^1^, Ravi S. Nanjundiah^1,6^, M. Rajeevan^8^

^1^Indian Institute of Tropical Meteorology, Ministry of Earth Sciences, India

^2^National Center for Atmospheric Research, Boulder, CO, 80301, USA

^3^Dept. of Atmospheric and Space sciences, Savitribai Phule Pune University, Pune, India

^4^India Meteorological Department, New Delhi, Ministry of Earth Sciences, India

^5^Centre for Development of Advanced Computing, Pune 411 008, India

^6^Centre for Atmospheric and Oceanic Sciences, Indian Institute of Science, Bengaluru 560 012, India

^7^National Center for Medium Range Weather Forecasting, Ministry of Earth Sciences, Noida, UP, India

^8^Ministry of Earth Sciences, Prithvi Bhavan, Lodhi Road, New Delhi 110003, India

*[email: chinmayjena@tropmet.res.in](mailto:email:%20chinmayjena@tropmet.res.in) and sachinghude@tropmet.res.in


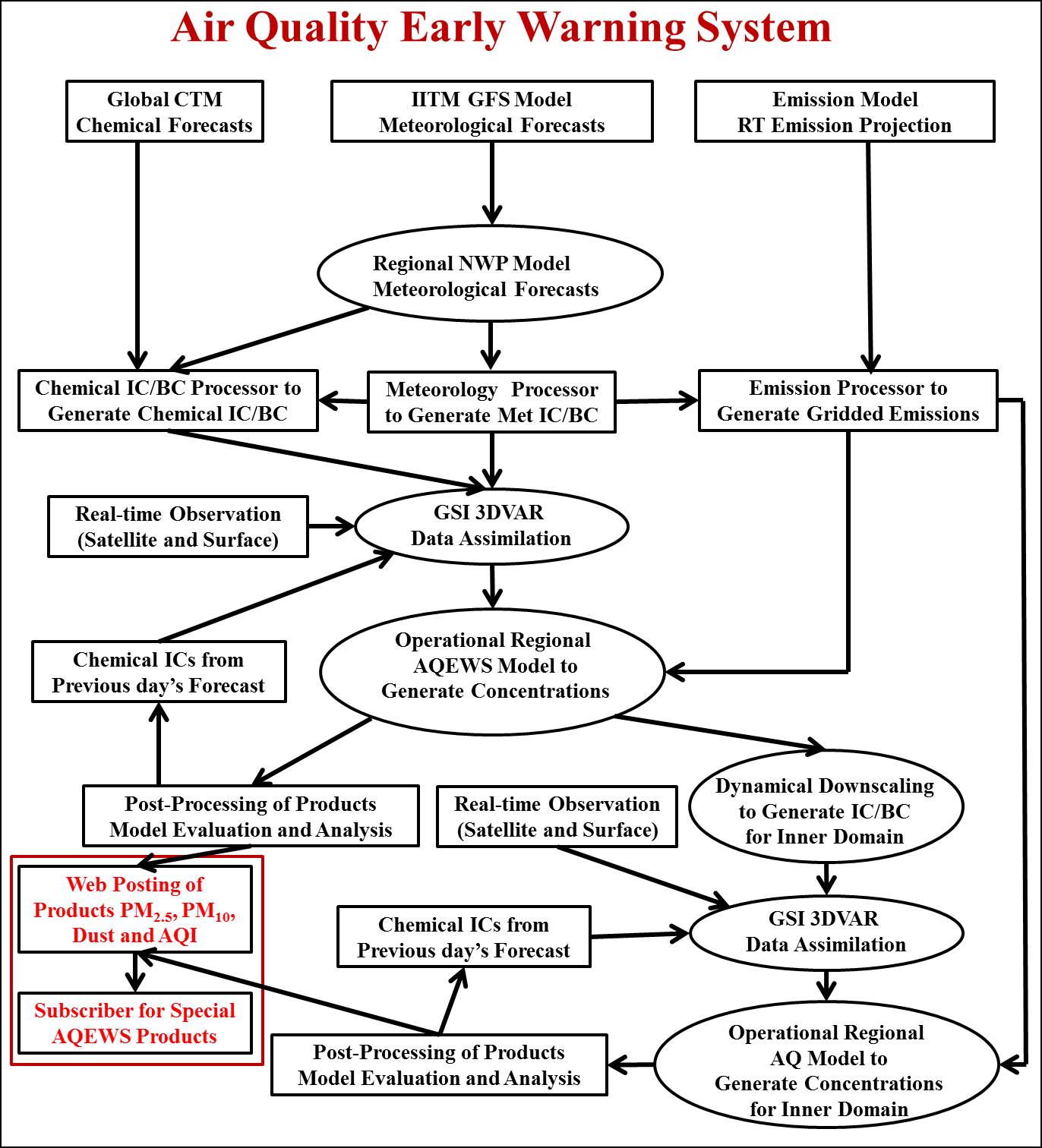


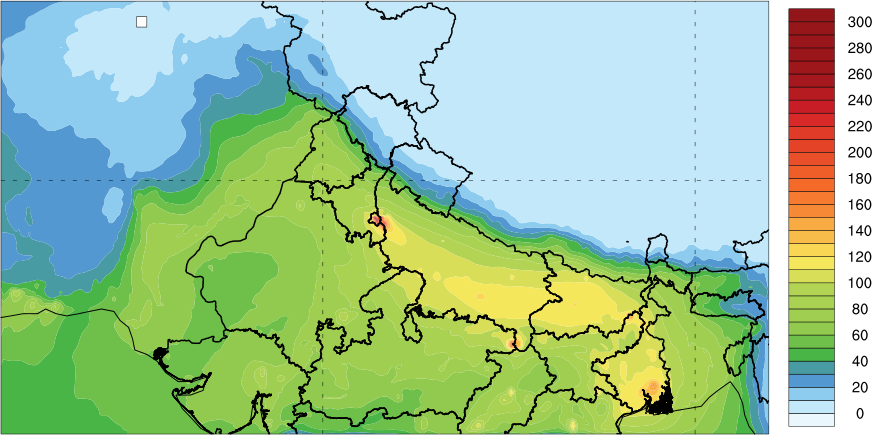

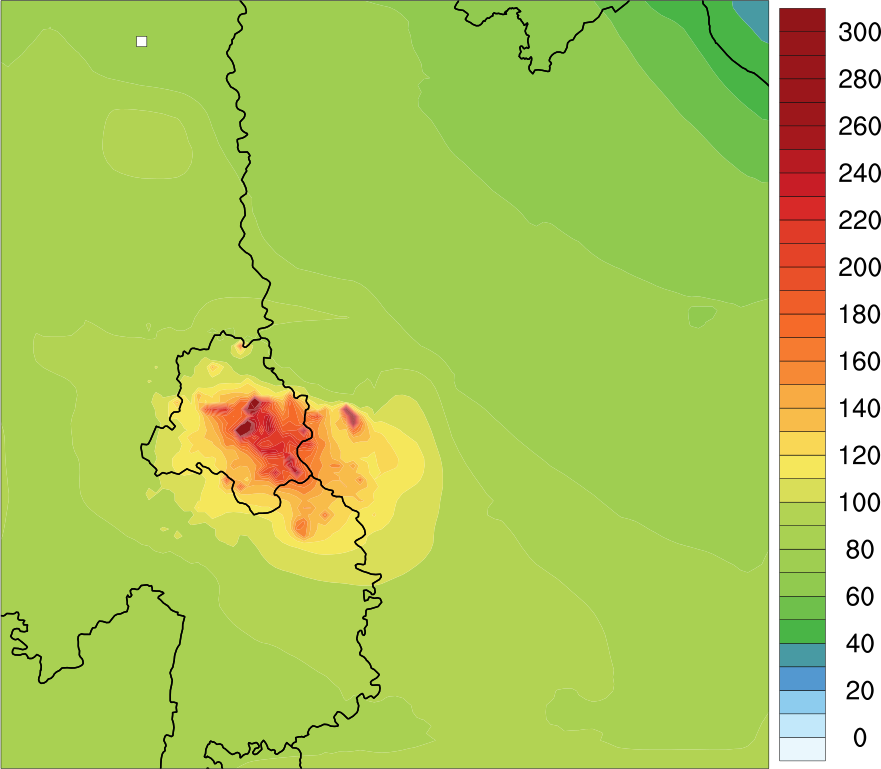

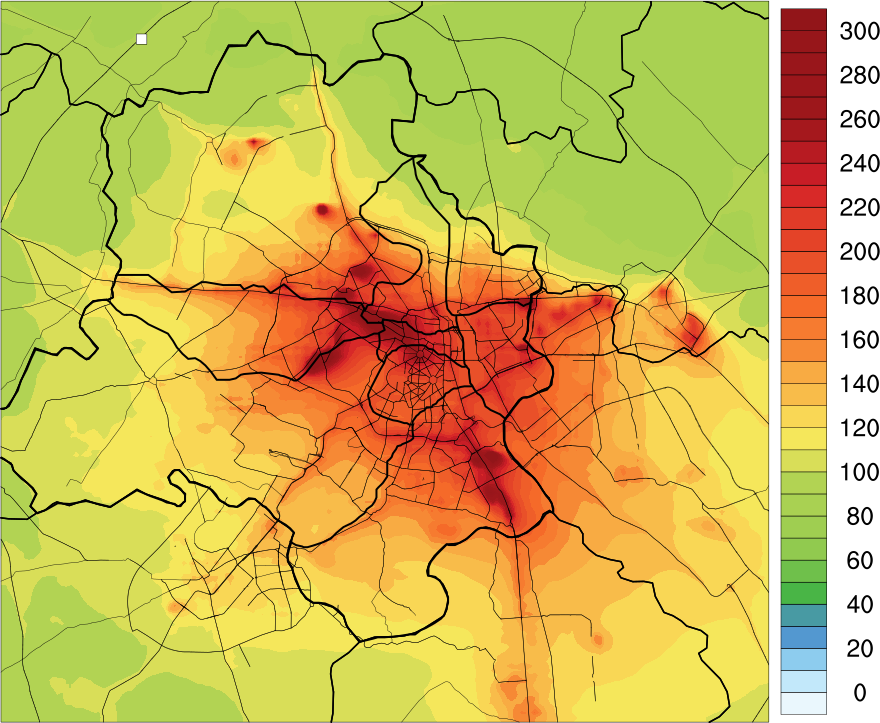


**D1**

**D2**

**D3**

**Figure S1:** Architecture of the very-high resolution air quality early warning system.


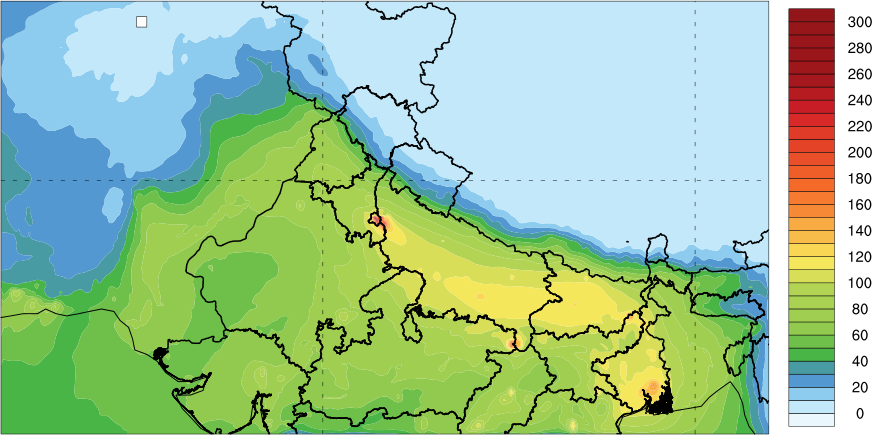

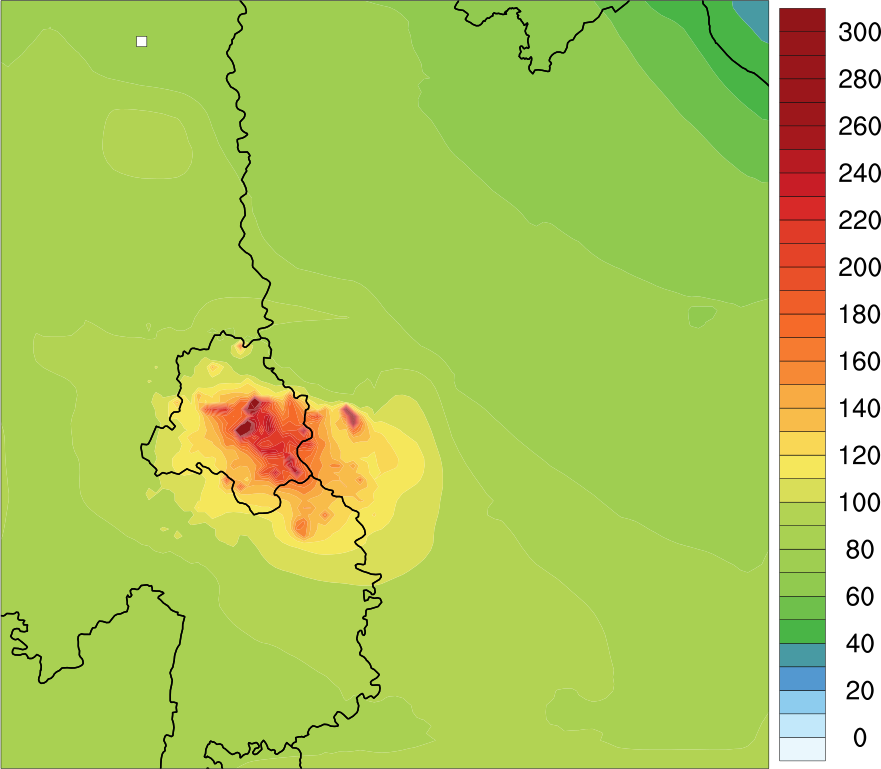

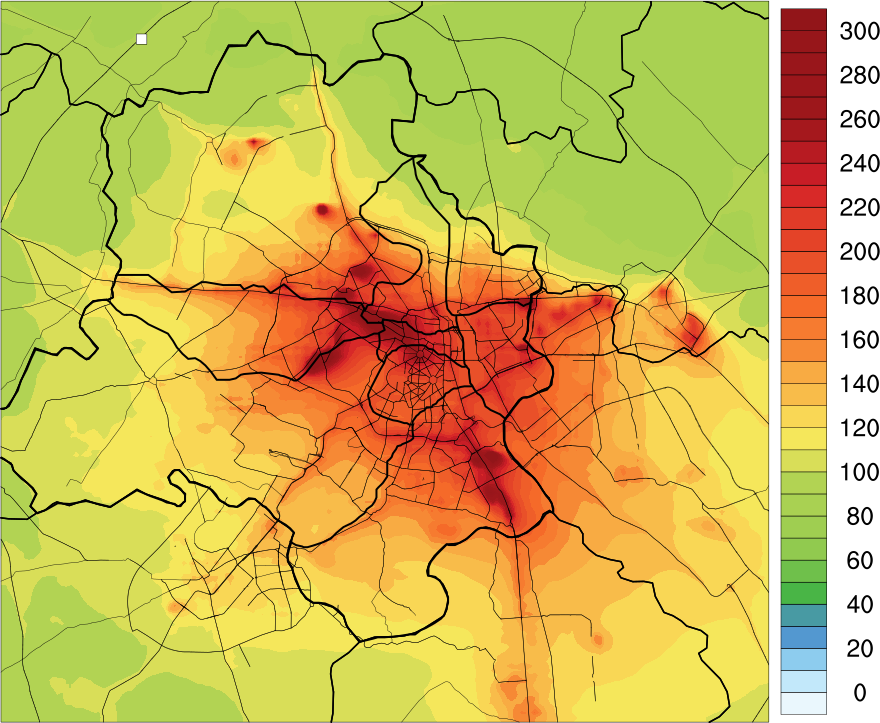


**D1**

**D2**

**D3**

**Figure S2:** Map of model simulation domain (D1: 10 km horizontal grid spacing, D2: 2 km horizontal grid spacing and D3: 400 meter horizontal grid spacing). We have used ncl/6.6.2 software to create the images (https://www.ncl.ucar.edu/).


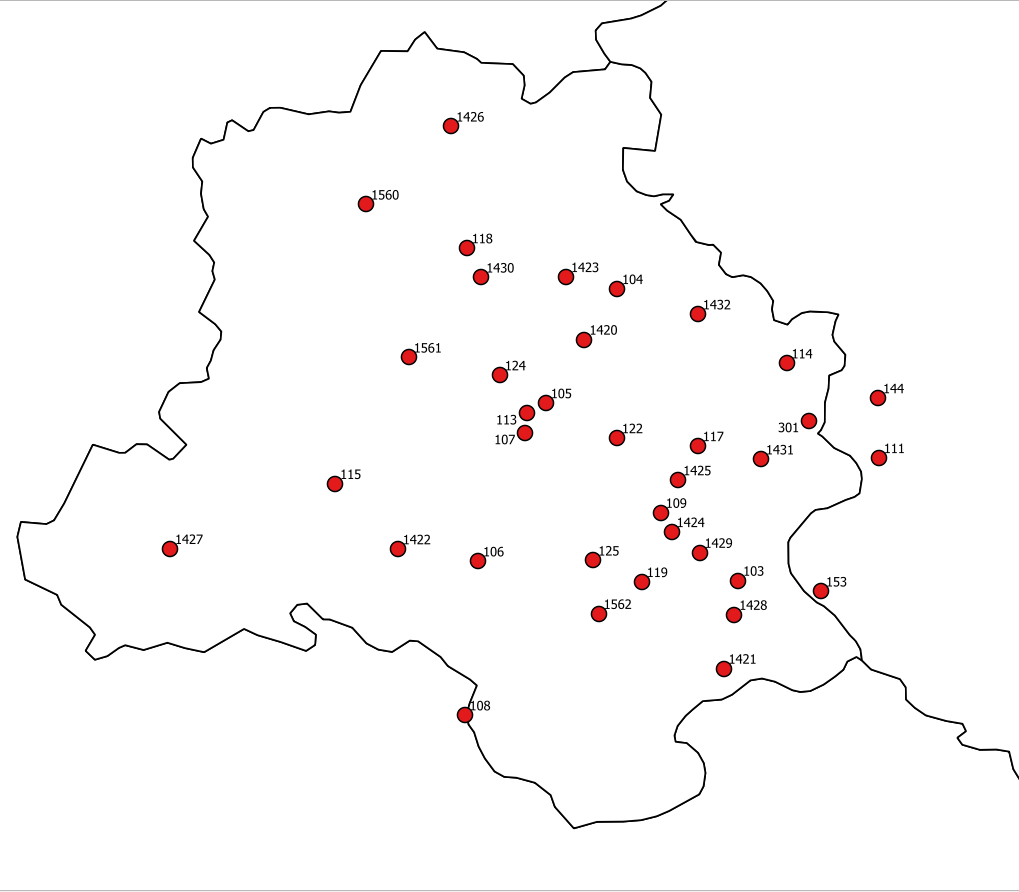


**Figure S3:** Geographical locations of 37 air quality monitoring stations (stations names associated with the numbers are provided in Table ST2)


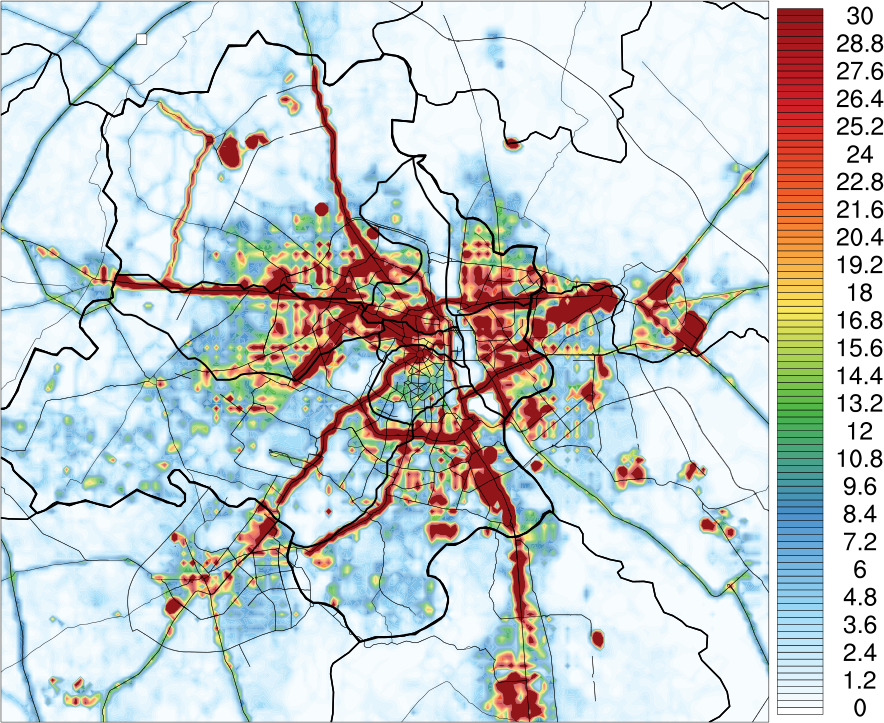


**Figure S4:** Spatial distribution of High-resolution Delhi Emission Inventory (HrDEI) of PM_2.5_ anthropogenic emissions (unit: 10^10^ kg/m^2^/s) at 400 m horizontal resolution. We have used ncl/6.6.2 software to create the image (https://www.ncl.ucar.edu/).


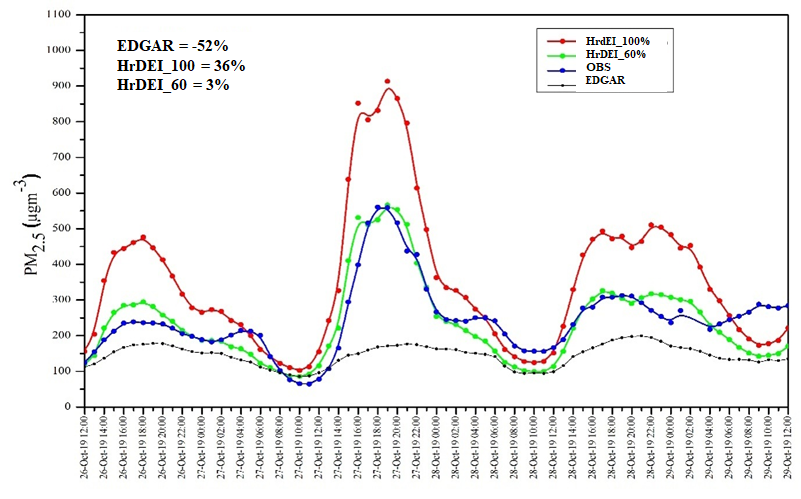


**Figure S5:** Sensitivity simulations for different emission inventory over Delhi.


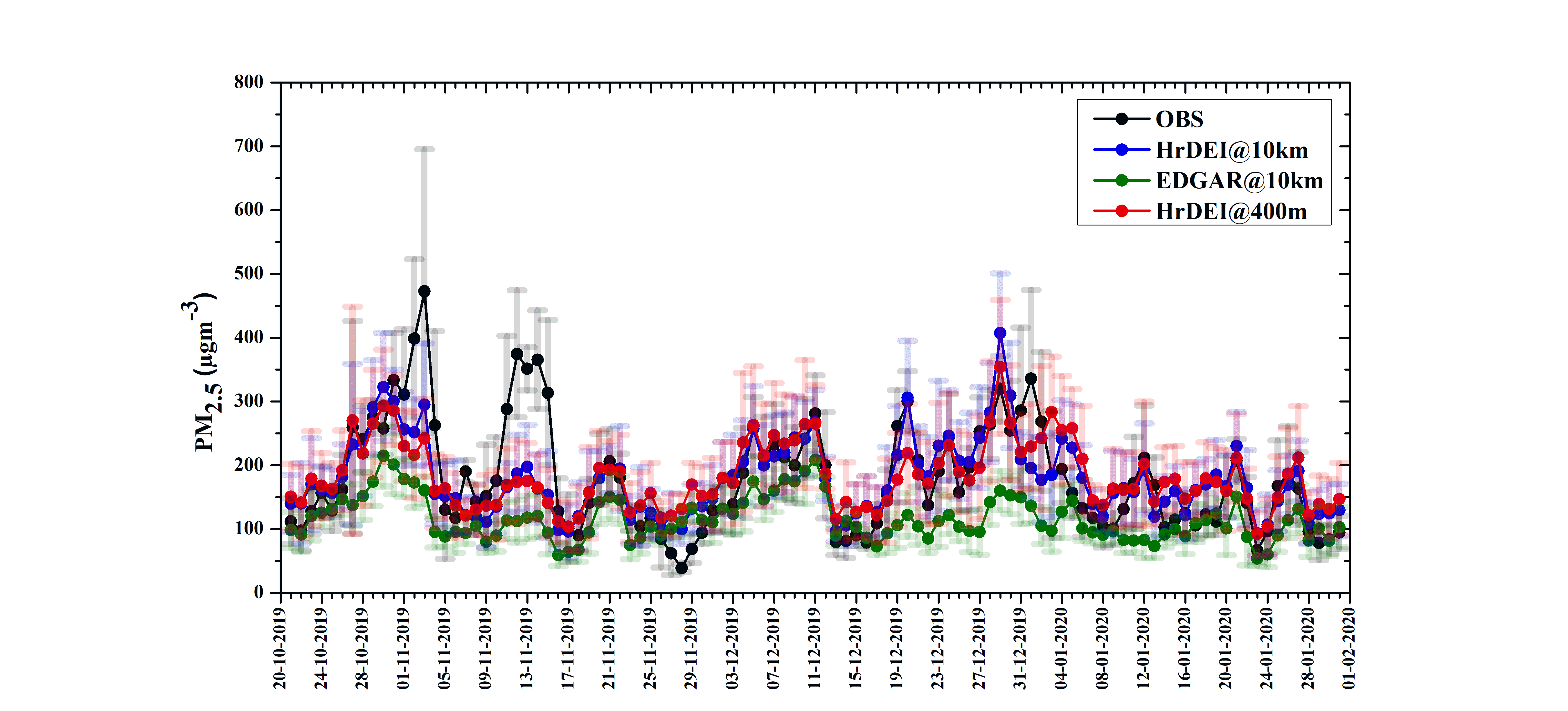


**Figure S6**: Time-series of daily surface-level PM_2.5_ concentrations from a) observations (black line) (average of 37 stations across Delhi) b) model simulations with EDGAR emissions inventory and with 10 km x 10 km grid-spacing (green line) c) model simulations with High-resolution Delhi Emission Inventory (HrDEI) and with 10 km x 10 km grid-spacing (blue line) and d) model simulations with HrDEI emissions’ inventory and with 400 m x 400 m grid-spacing (red line) of 1^st^ day forecast.


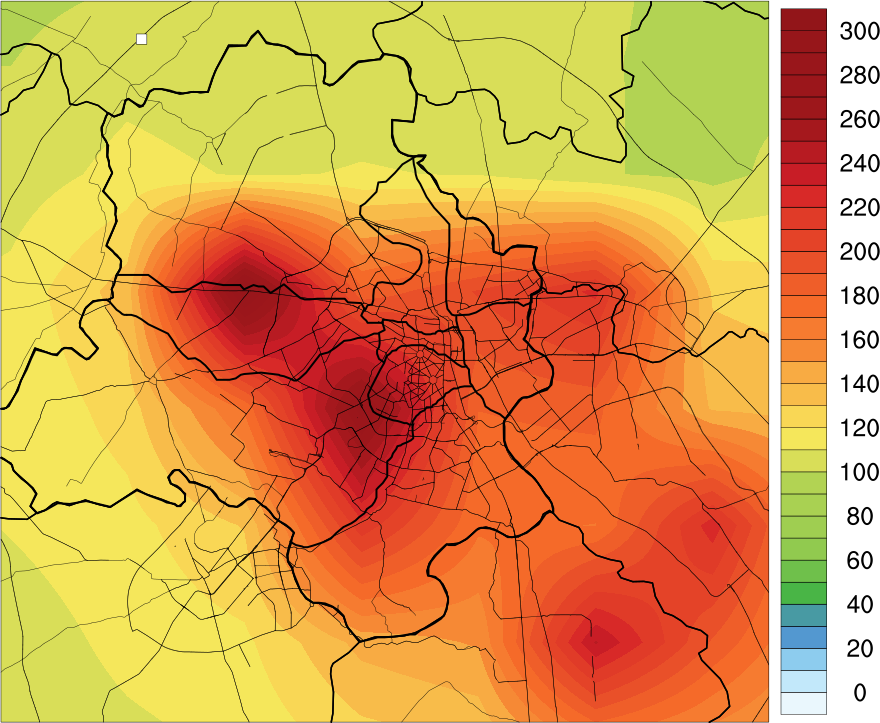

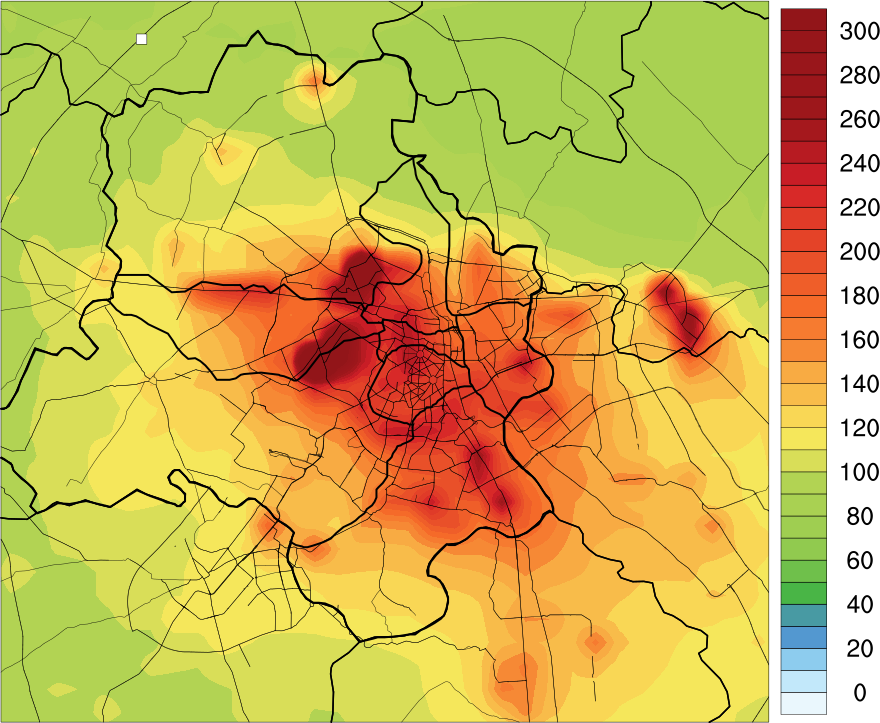


**10KM**

**2KM**

**Figure S7:** Spatial distribution of average PM_2.5_ of 1^st^ day forecast for 400 meter grid spacing during 21 October 2019 to 01 February 2020 at 10 km grid spacing (left) and 2 Km Horizontal grid spacing (right). We have used ncl/6.6.2 software to create the images (https://www.ncl.ucar.edu/).


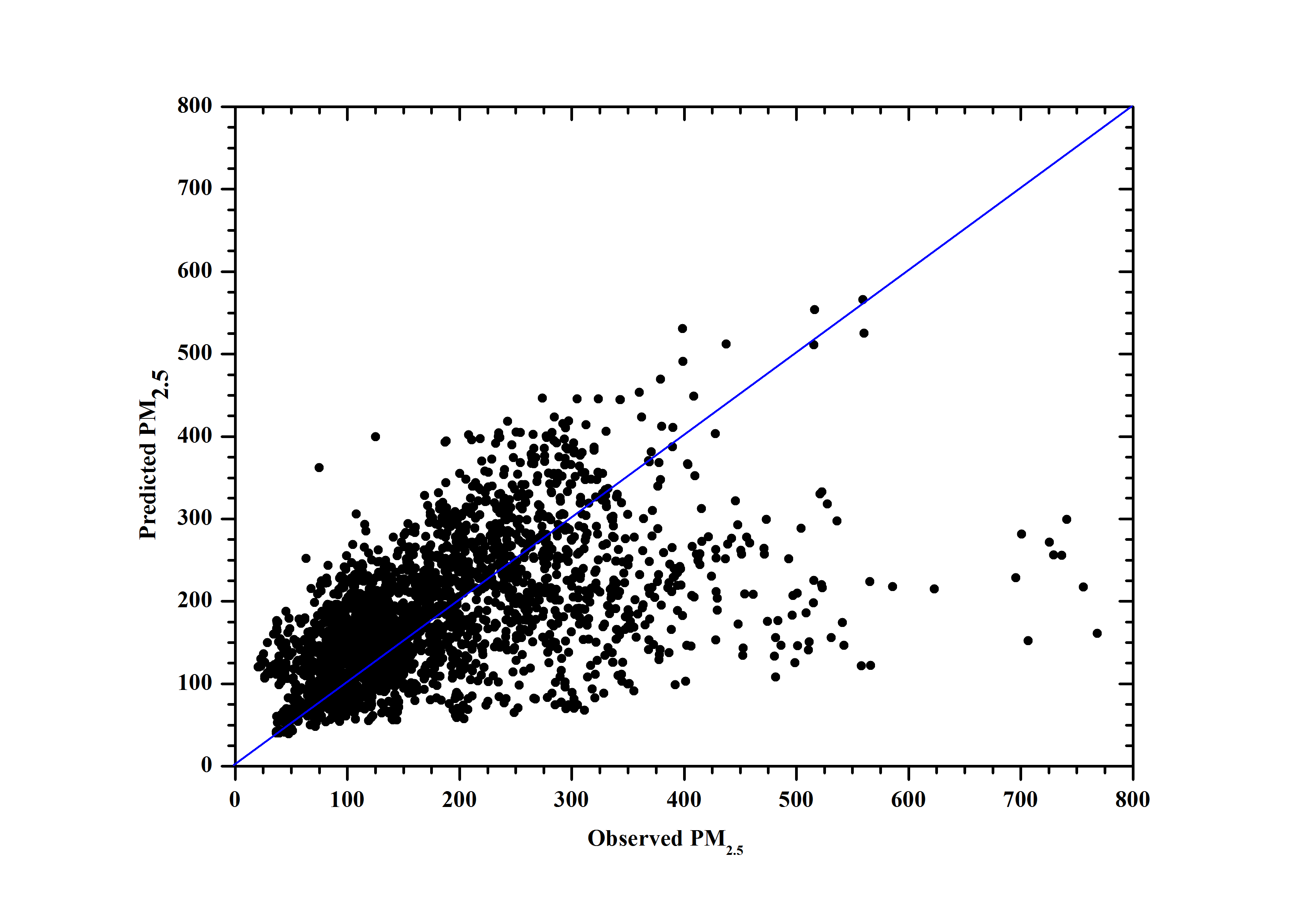


**Figure S8:** Correlation between hourly mean observed and predicted PM_2.5_ in Delhi.

**Table ST1:** Selected atmospheric physical and chemical parameterizations

| **Atmospheric Process** | **Parameterization** |
| --- | --- |
| Cloud Microphysics | WRF Single-Moment 6-class scheme (WSM6)^1^ |
| Short- and Long-wave radiation | Rapid Radiative Transfer Model for GCMs^2^ |
| Surface Layer | Monin-Obukhov (Janjic Eta)Scheme^3,4^ |
| Land Surface model | Unified Noah Land-surface model^5^ |
| Planetary Boundary Layer | MYNN2.5^6^ |
| Cumulus | Grell-Freitas ensemble scheme^7^ |
| Gas-phase Chemistry | Model for Ozone and Related Tracers^8^ |
| Aerosol Processes | Goddard Global Ozone Chemistry Aerosol Radiation and Transport (GOCART)^9^ |

**Table ST2**: Performance statistics for mean PM2.5 forecast for different emission inventory and different grid spacing of 1st day forecast over Delhi.

| **State**  **Variables** | **Emission Variables** | **MB** | **NMFB (%)** | **NMFE (%)** | **r** |
| --- | --- | --- | --- | --- | --- |
| **PM25_hourly** | **EDGAR@10km** | **-63.1** | **-42.6** | **51.1** | **0.5** |
|  | **HrDEI@10km** | **-0.3** | **-0.2** | **33.1** | **0.6** |
|  | **HrDEI@400m** | **2.5** | **1.3** | **36.3** | **0.5** |
| **PM25_daily** | **EDGAR@10km** | **-64.9** | **-43.8** | **47.8** | **0.5** |
|  | **HrDEI@10km** | **-1.0** | **-0.5** | **23.2** | **0.7** |
|  | **HrDEI@400m** | **1.8** | **1.0** | **25.6** | **0.6** |

**Table ST3:** Performance statistics for simulated PM_2.5_ at different monitoring sites in Delhi during 21 October 2019 to 01 February 2020 at 400 m horizontal grid-spacing.

| State | Station name | Latitude | Longitude | MB | NMB (%) | RMSE | R |
| --- | --- | --- | --- | --- | --- | --- | --- |
| Delhi | CRRI Mathura Road (103) | 28.5512005 | 77.2735737 | 158.7 | 87.6 | 249.9 | 0.4 |
|  | Burari Crossing (104) | 28.7256504 | 77.2011573 | -60.2 | -31.0 | 149.5 | 0.3 |
|  | North Campus DU (105) | 28.6573814 | 77.1585447 | 68.4 | 40.8 | 181.6 | 0.3 |
|  | IGI-Airport-T3 (106) | 28.5627763 | 77.1180053 | -12.6 | -8.1 | 106.0 | 0.4 |
|  | Pusa IMD (107) | 28.639645 | 77.146262 | 115.2 | 83.4 | 208.1 | 0.3 |
|  | DTU (118) | 28.7500499 | 77.1112615 | -95.6 | - 45.6 | 161.2 | 0.3 |
|  | R K Puram (124) | 28.674045 | 77.131023 | 59.4 | 37.2 | 154.0 | 0.4 |
|  | Shadipur (113) | 28.6514781 | 77.1473105 | 104.4 | 65.5 | 177.0 | 0.6 |
|  | NSIT Dwarka (115) | 28.60909 | 77.0325413 | -36.1 | - 20.7 | 92.4 | 0.5 |
|  | Mandir Marg (122) | 28.636429 | 77.201067 | 52.2 | 29.1 | 174.3 | 0.2 |
|  | Punjabi Bagh (125) | 28.563262 | 77.186937 | -0.5 | - 0.2 | 122.1 | 0.5 |
|  | Sirifort (119) | 28.5504249 | 77.2159377 | -12.4 | - 6.3 | 120.4 | 0.4 |
|  | Lodhi Road (109) | 28.5918245 | 77.2273074 | 34.4 | 23.3 | 127.8 | 0.3 |
|  | ITO (117) | 28.6316945 | 77.2494387 | 20.0 | 10.7 | 144.4 | 0.3 |
|  | Anand Vihar (301) | 28.646835 | 77.316032 | -52.3 | - 25.0 | 145.2 | 0.5 |
|  | Sector – 62 (111) | 28.6245479 | 77.3577104 | -33.5 | -17.3 | 133.8 | 0.3 |
|  | IHBAS-Dilshad-Garden (114) | 28.6811736 | 77.3025234 | 13.3 | 8.5 | 118.5 | 0.4 |
|  | Aya Nagar (108) | 28.4706914 | 77.1099364 | -23.8 | -15.7 | 110.8 | 0.4 |
|  | Vasundhara (144) | 28.6603346 | 77.3572563 | -31.5 | - 14.0 | 140.5 | 0.4 |
|  | Sector 125 (153) | 28.5447608 | 77.3231257 | -14.9 | - 7.5 | 140.9 | 0.3 |
|  | Ashok_Vihar (1420) | 28.695381 | 77.181665 | 26.1 | 24.3 | 79.0 | 0.2 |
|  | DKSS_Stadium (1421) | 28.498571 | 77.264840 | -48.0 | - 24.5 | 137.8 | 0.3 |
|  | Dwarka Sector8 (1422) | 28.57 | 77.07 |  |  |  |  |
|  | Jahangirpuri (1423) | 28.732820 | 77.170633 | -81.1 | - 35.9 | 151.5 | 0.3 |
|  | Jawaharlal Nehru Stadium (1424) | 28.580280 | 77.233829 | 3.0 | 1.5 | 116.8 | 0.5 |
|  | MDC National Stadium (1425) | 28.611281 | 77.237738 | 32.7 | 19.2 | 140.0 | 0.3 |
|  | Najafgarh (1427) | 28.570173 | 76.933762 | 46.1 | 85.3 | 59.3 | 0.1 |
|  | Narela (1426) | 28.822836 | 77.101981 | -49.4 | - 38.8 | 66.8 | 0.1 |
|  | Nehru Nagar (1429) | 28.567890 | 77.250515 | -9.2 | - 3.8 | 145.3 | 0.5 |
|  | Okhla Phase2 (1428) | 28.530785 | 77.271255 | 17.4 | 14.7 | 77.6 | 0.2 |
|  | Patparganj (1431) | 28.623748 | 77.287205 | 28.7 | 16.0 | 122.5 | 0.4 |
|  | Rohini (1430) | 28.732528 | 77.119920 | -88.6 | - 39.1 | 163.0 | 0.4 |
|  | Sonia Vihar (1432) | 28.710508 | 77.249485 | -44.6 | - 25.8 | 114.3 | 0.3 |
|  | Sri_Aurbindo_Marg (1562) | 28.531346 | 77.190156 | 2.6 | 3.1 | 53.5 | 0.1 |
|  | Mundak (1561) | 28.684678 | 77.076574 | 19.0 | 18.8 | 50.8 | 0.6 |
|  | New_collectorate (1569) | 28.974801 | 77.213357 | -83.3 | - 46.4 | 142.2 | 0.4 |
|  | New_mandi (1550) | 29.4723508 | 77.7194031 | -65.0 | - 44.9 | 109.6 | 0.4 |
|  | Bawana (1560) | 28.776200 | 77.051074 | -92.6 | - 41.9 | 163.4 | 0.4 |

**Table ST4:** Model performance goals used to evaluate the model performance for PM_2.5_ (Morris et al., 2005)

| **Fractional Bias** | **Fractional Error** | **Comment** |
| --- | --- | --- |
| ≤ ± 15% | ≤ 35% | A level of model performance that would be considered excellent |
| ≤ ± 30% | ≤ 50% | A level of model performance that would be considered good |
| ≤ ± 60% | ≤ 75% | A level of model performance that would be considered average and hope each PM species could meet for regulatory modeling |
| > ± 60% | > 75% | At or exceeding this level of performance indicates fundamental problems with the modeling system |

**Table ST5:** AQI category and corresponding break-point concentrations ranges for PM_2.5_ based on National Ambient Air Quality Standard (NAAQS)**.**

| **AQI Category** | **AQI** | **PM_2.5_**  **Concentration range** |
| --- | --- | --- |
| **Good** | **0 - 50** | **0 - 30** |
| **Satisfactory** | **51 - 100** | **31 - 60** |
| **Moderately** | **100 - 200** | **61 - 90** |
| **Poor** | **201 - 300** | **91 - 120** |
| **Very poor** | **301 - 400** | **121 - 250** |
| **Severe** | **401 +** | **250+** |

| State | PM_25_ AQI  Category | Variables | **10km** | | | **2km** | | | **400 meter** | | |
| --- | --- | --- | --- | --- | --- | --- | --- | --- | --- | --- | --- |
|  |  |  | **MB** | **NMFB (%)** | **NMFE (%)** | **MB** | **NMFB (%)** | **NMFE (%)** | **MB** | **NMFB (%)** | **NMFE (%)** |
| Delhi | Poor  (201-300) | 1^st^ day | 51.1 | 18.4 | 19.3 | 66.2 | 23.2 | 23.3 | 62.9 | 22.1 | 22.3 |
|  |  | 2^nd^ day | 30.4 | 11.4 | 20.8 | 56.1 | 20.0 | 22.6 | 53.6 | 19.2 | 22.2 |
|  |  | 3^rd^ day | 16.3 | 6.2 | 23.2 | 42.4 | 15.4 | 20.9 | 44.6 | 16.2 | 20.2 |
|  | Very Poor  (301-400) | 1^st^ day | 4.2 | 1.2 | 6.4 | 12.3 | 3.5 | 7.4 | 8.2 | 2.3 | 6.8 |
|  |  | 2^nd^ day | -17.7 | -5.3 | 9.1 | 0.2 | 0.1 | 6.7 | -2.7 | -0.8 | 6.9 |
|  |  | 3^rd^ day | -27.5 | -8.3 | 11.4 | -13.7 | -4.0 | 8.9 | -13.4 | -3.9 | 8.7 |
|  | Severe  (401-above) | 1^st^ day | -47.1 | -11.1 | 15.6 | -55.5 | -13.3 | 16.2 | -58.0 | -13.9 | 16.3 |
|  |  | 2^nd^ day | -89.0 | -22.1 | 22.2 | -70.2 | -17.1 | 17.5 | -70.8 | -17.2 | 17.8 |
|  |  | 3^rd^ day | -105.0 | -26.7 | 26.7 | -86.2 | -21.4 | 21.8 | -83.6 | -20.7 | 20.9 |

**Table ST6:** Performance statistics of different PM_2.5_ AQI forecast category

**
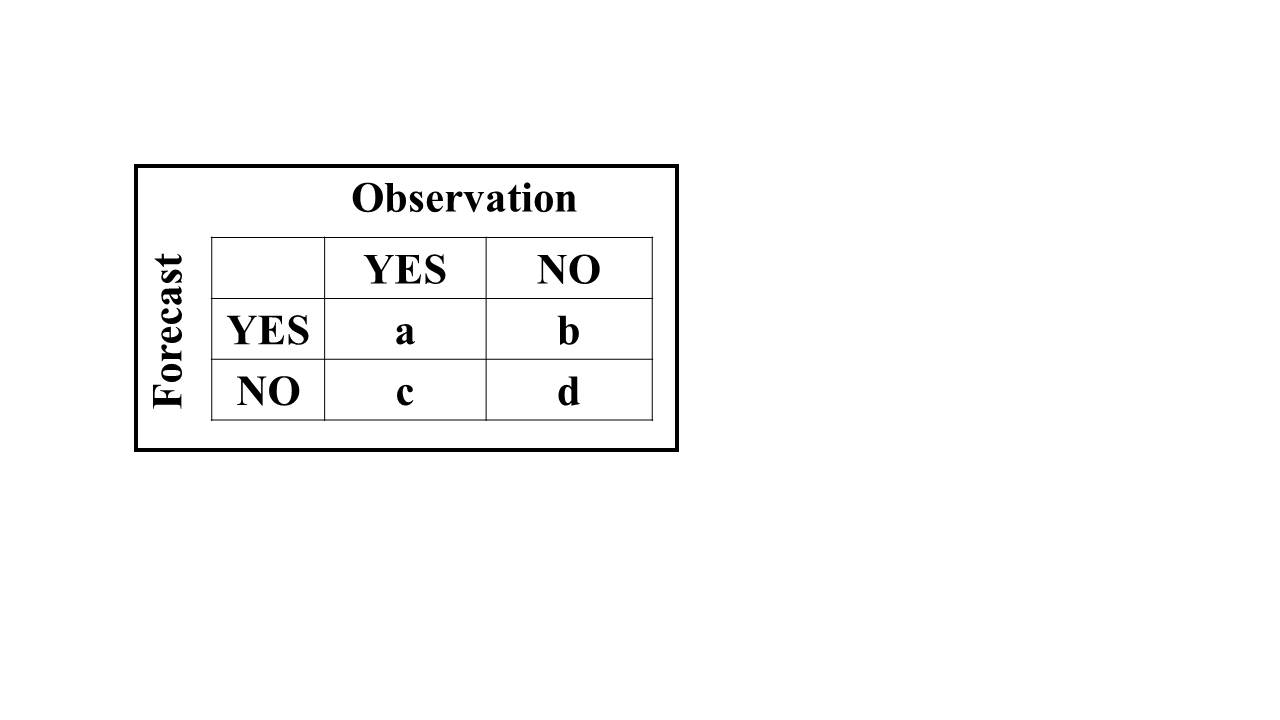
Table ST7:** A contingency table and equations used to calculate the different skill score for different category of AQI forecast.

| Statistic name | What it measures | Equation | unit | How to interpret |
| --- | --- | --- | --- | --- |
| **Accuracy (A)** | Percent of forecasts that correctly predicted the event or non-event. | A=(a+d)/(a+b+c+d) *100 | % | Higher numbers are better |
| **False Alarm Rate (FAR)** | The percent of times a forecast of high pollution did not actually occur. | FAR = (b/(a+b)) *100 | % | Smaller values are best |
| **Probability of Detection**  **(POD) or Hit rate** | Ability to predict high pollution events (i.e., the percentage of forecasted high pollution events that actually occurred). | POD = (a/(a+c)) * 100 | % | Higher numbers are best |
| **Critical Success Index**  **(CSI), also called Threat**  **Score** | How well the high-pollution events were predicted. Useful for evaluating rarer events like high-pollution days. It is not affected by a large number of correctly forecasted, low pollution events. | CSI = (a/(a+b+c)) * 100 | % | Higher numbers are best |

**Reference:**

1. Hong, S.-Y. and Lim, J.-O. J. 2006. The WRF single-moment 6-class microphysics scheme (WSM6). J. Kor. Meteorol. Soc. 42, 129-151.
2. Iacono M.J., J.S. Delamere, E.J. Mlawer, M.W. Shephard, S.A. Clough, and W. Collins, Radiative forcing by long-lived greenhouse gases: Calculations with the AER radiate transfer models, J. Geophys. Res., 113, D13103, doi:10.1029/2008JD009944, 2008.
3. Janjic, Z. I., 1996: The surface layer in the NCEP Eta Model, Eleventh Conference on Numerical Weather Prediction, Norfolk, VA, 19–23 August;Amer. Meteor. Soc., Boston, MA, 354–355.
4. Janjic, Z. I., 2002: Nonsingular Implementation of the Mellor–Yamada Level 2.5 Scheme in the NCEP Meso model, NCEP Office Note, No. 437, 61.
5. Tewari, M., Chen, F., Wang, W., Dudhia, J., Lemone, M. A., Mitchell, K. E. (2004). Implementation and verification of the unified Noah land-surface model in the WRF model [presentation]. In 20th Conference on Weather Analysis and Forecasting/16th Conference on Numerical Weather Prediction. American Meteorological Society: Seattle, WA, US.
6. Nakanishi, M., and H. Niino, 2006: An improved Mellor–Yamada level-3 model: Its numerical stability and application to a regional prediction of advection fog. Bound. Layer Meteor., 119, 397–407.
7. Grell, G. A., & Freitas, S. R. (2014). A scale and aerosol aware stochastic convective parameterization for weather and air quality modelling, Atmospheric Chemistry and Physics, 14, 5233–5250. https://doi.org/10.5194/acp-14-5233-2014
8. Emmons, L. K., Walters, S., Hess, P. G., Lamarque, J.-F., Pfister, G. G., Fillmore, D., Granier, C., Guenther, A., Kinnison, D., Laepple, T., Orlando, J., Tie, X., Tyndall, G., Wiedinmyer, C., Baughcum, S. L., and Kloster, S.: Description and evaluation of the Model for Ozone and Related chemical Tracers, version 4 (MOZART-4), Geosci. Model Dev., 3, 43–67, 2010.
9. Chin, M., Savoie, D. L., Huebert, B. J., Bandy, A. R., Thornton, D. C., Bates, T. S., Quinn, P. K., Saltzman, E. S., and De Bruyn, W. J.: Atmospheric sulfur cycle simulated in the global model GOCART: Comparison with field observations and regional budgets, J. Geophys. Res.-Atmos., 105, 24689–24712, https://doi.org/10.1029/2000JD900385, 2000.
